# First identification of Cryptosporidium parvum virus 1 (CSpV1) in various subtypes of *Cryptosporidium parvum* from diarrheic calves, lambs and goat kids from France

**DOI:** 10.1186/s13567-023-01196-4

**Published:** 2023-08-22

**Authors:** Karim Tarik Adjou, Aurélie Chevillot, Pierrick Lucas, Yannick Blanchard, Houria Louifi, Razika Arab, Mohamed Mammeri, Myriam Thomas, Bruno Polack, Grégory Karadjian, Nolwenn M. Dheilly

**Affiliations:** 1 Laboratoire de Santé Animale, Ecole Nationale Vétérinaire d’Alfort, Anses, INRAE, UMR BIPAR, 94700 Maisons-Alfort, France; 2 Anses Animal Health Laboratory, UMR1161 Virology, INRAE, Anses, ENVA, Maisons-Alfort, France; 3 Laboratoire de Ploufragan-Plouzané-Niort, Unité Génétique virale et biosécurité, ANSES, Agence Nationale de Sécurité Sanitaire de l’Alimentation, de l’Environnement et du Travail, Ploufragan, France

**Keywords:** *Cryptosporidium parvum*, cryspovirus, calves, lambs, kids, France

## Abstract

*Cryptosporidium* spp. remain a major cause of waterborne diarrhea and illness in developing countries and represent a significant burden to farmers worldwide. Cryptosporidium parvum virus 1 (CSpV1), of the genus *Cryspovirus*, was first reported to be present in the cytoplasm of *C. parvum* in 1997. Full-length genome sequences have been obtained from *C. parvum* from Iowa (Iowa), Kansas (KSU) and China. We aimed at characterizing the genome of CSpV1 from France and used sequence analysis from *Cryptosporidium* isolates to explore whether CSpV1 genome diversity varies over time, with geographical sampling location, *C. parvum* genetic diversity, or ruminant host species. A total of 123 fecal samples of cattle, sheep and goats were collected from 17 different French departments (57 diseased animal fecal samples and 66 healthy animal fecal samples). Subtyping analysis of the *C. parvum* isolates revealed the presence of two zoonotic subtype families IIa and IId. Sequence analysis of CSpV1 revealed that all CSpV1 from France, regardless of the subtype of *C. parvum* (IIaA15G2R1, IIaA17G2R1 and IIdA18G1R1) are more closely related to CSpV1 from Turkey, and cluster on a distinct branch from CSpV1 collected from *C. parvum* subtype IIaA15G2R1 from Asia and North America. We also found that samples collected on a given year or successive years in a given location are more likely to host the same subtype of *C. parvum* and the same CSpV1 strain. Yet, there is no distinct clustering of CSpV1 per French department or ruminants, probably due to trade, and transmission of *C. parvum* among host species. Our results point towards (i) a close association between CSpV1 movement and *C. parvum* movement, (ii) recent migrations of *C. parvum* among distantly located departments and (iii) incidental transmission of *C. parvum* between ruminants. All together, these results provide insightful information regarding CSpV1 evolution and suggest the virus might be used as an epidemiological tracer for *C. parvum*. Future studies need to investigate CSpV1’s role in *C. parvum* virulence and on subtype ability to infect different species.

**Supplementary Information:**

The online version contains supplementary material available at 10.1186/s13567-023-01196-4.

## Introduction

Species of the genus *Cryptosporidium* are protozoan parasites specific of vertebrates that are responsible for mild to severe diarrhea in both human and animals [1, 2]. In ruminants, diarrhea is particularly severe in newborn and young. It causes dehydration, weakness, anorexia, weight-loss and a delayed growth. Adults can remain asymptomatic and serve as reservoir host for the parasite [3]. Thus, Cryptosporidiosis represents a significant economic loss for farmers. *Cryptosporidium* is also a public health issue because contamination of surface water can lead to human infections. *Cryptosporidium* is among the most important cause of diarrhea in developing countries, and remain a major cause of waterborne outbreaks and illness worldwide in both immunocompetent and immunocompromised people. In healthy immunocompetent people, cryptosporidiosis usually resolves itself within a couple of weeks [4–7]. However, epidemic outbreaks are still associated with fatalities, mostly in young children, and a chronic and life-threatening cryptosporidiosis can develop in immunocompromised individuals, such as those affected by HIV, transplant recipients or infants [7].

The genus *Cryptosporidium* is composed of 44 recognized species, and 120 genotypes remain of unknown species status [8]. They represent different public health significance depending on their host specificity and zoonotic potential. Humans are mainly infected by *C. parvum* and *C. hominis*. *C. parvum* is by far the *Cryptosporidium* species with the broadest host range and most important zoonotic potential. Nearly 20 *C. parvum* subtypes have been described using the 60 kDa glycoprotein gene (*gp60*) as genetic locus. The subtype family IIc appears to have adapted to humans whereas the subtype family IIa is mostly found in cattle and subtype family IId is mostly found in sheep and goats [9, 10]. The highly transmissible subtype IIaA15G2R1 is the most prevalent in most industrialized nations [10–12]. In France, this highly transmissible subtype has been reported in feces from cattle, goat and sheep [13–15]. However, *C. parvum* has a panmictic/epidemic population structure resulting in a high genetic diversity, with frequent disagreement between *gp60* results and other genetic markers due to frequent genetic recombination within this subtype [11]. Multilocus typing tools have allowed a better characterization of *C. parvum* subtype IIaA15G2R1, revealing a geographic segregation with country-specific clusters and a genetic distance that correlates with geographic distance [16, 17]. Different subpopulations have also been found in different host species due to reduced gene flow [18–20]. The global expansion of the IIaA15G2R1 zoonotic subtype presents a major challenge. In particular, there is a need to develop novel, easy to use methods to track *C. parvum’s* genetic structure, host-adaptation, virulence and threat to public health.

The C. parvum virus 1, of the genus *Cryspovirus*, family *Partitiviridae*, was first reported in *C. parvum* cytoplasm in 1997 [21, 22]. The viral genome is bi-segmented and dsRNA1 and dsRNA2 each encode a single protein. Closely related strains of the cryspovirus were found in distinct *C. parvum* strains infecting humans and calves, as well as in *C. hominis*, *C. felis* and *C. meleagridis*, with > 92% amino-acid similarities [23, 24]. A single study has assessed the cryspovirus prevalence in parasite populations, in Japan [25]. It showed that CSpV1 has 100% apparent prevalence and appears to co-diversify with *C. parvum* so that viral sequences might be used for sensitive detection of *C. parvum subtype* IIaA15G2R1 and to determine the regional origin of the parasite [25–27].

The aims of the present study were the following: (1) to determine by PCR (*18S rRNA*, PCR–RFLP, *gp60*) the presence of *Cryptosporidium* in calves, lambs and kid goats in different regions of France, (2) to characterize the presence and prevalence of cryspovirus in these French *Cryptosporidium* isolates, (3) to genotype the virus in order to test whether CSpV1 genome diversity varies over time, with geographical sampling location, with *C. parvum* genetic diversity, or with ruminant host species.

## Materials and methods

### Sample collection

A total of 123 fecal samples were collected voluntarily by veterinarians or departmental veterinarian laboratories, between 2018 and 2022, and originated from 17 different French departments (Table 1). Among those, 57 samples were collected from diseased animals that had been naturally infected by *Cryptosporidium* and presented a diarrhea at the time of sampling. An additional 66 fecal samples were collected from apparently healthy animals. Samples were collected with sterile gloves, mailed at room temperature, and conserved at 4 °C until use.

**Table 1:** **Number and origin of samples used in this study.**

Departement of origin	Sheep		Cattle		Goats	
	Diseased lambs	Healthy	Diseased calves	Healthy	Diseased Goat kids	Healthy
Tarn (81)	15	–	–	–	4	–
Haute-Vienne(87)	7	–	–	–	–	–
Loiret (45)	1	9 adults	–	–	–	–
Loire (42)	–	8 lambs	5	–	–	–
Allier (3)	–	-	8	–	–	–
Puy-de-Dôme (63)	–	-	4	–	–	–
Moselle (57)	–	-	2	–	–	–
Nièvre (58)	–	2 adults	1	–	–	8 adults + 6 kids
Yonne (89)	–	6 lambs	–	–	–	-
Torcy (77)	–	16 adults	–	–	–	2 adults
Côte d’or (21)	–	4 adults	–	–	–	–
Cher (18)	–	1 adult	–	–	–	–
Aveyron (12)	–	1 adult	–	–	–	–
Aisne (2)	–	2 adults	–	–	–	–
Lot (64)	1	–	–	–	–	–
Pyrénées-Atlantiques (46)	6	–	–	–	–	–
Ardennes (08)	–	–	3	1		
Total	30	49	23	1	4	16

### Cryptosporidium detection and enrichment

*Cryptospridium* spp. were initially detected microscopically with direct immunofluorescence assay (DFA) (MeriFluor® *Cryptosporidium*/*Giardia*, Meridian Bioscience Europe, Milano, Italy) as previously described [14]. *Cryptosporidium* oocysts were purified from samples with positive DFA using Dynabeads™ anti-*Cryptosporidium* kit per manufacturer protocol. The success of *Cryptosporidium* purification was controlled using DFA.

### DNA extraction, *Cryptosporidium* species determination and *C. parvum* subtyping

Disruption of oocyst walls was achieved using ten freeze–thaw cycles as previously described [28]. Then, DNA extraction was conducted using the QIAamp DNA Stool Mini Kit (Qiagen, France), according to the manufacturer's instructions. The *Cryptosporidium* genus was determined using a nested PCR amplifying a 840 bp fragment of 18S rRNA gene [29]. PCR products were digested with *SspI* and *MboII* endonucleases and restriction fragment length polymorphism (RFLP) analysis was used to determine the *Cryptosporidium* species [30]. *C. parvum* samples were subtyped by nested PCR-sequence analysis of the partial 60 kDa glycoprotein locus (*gp60*) [31] and Sanger sequencing of both strands (Genoscreen) (Genbank numbers in Table 2). *C. parvum* subtypes were named using the recommended nomenclature system [10, 32].

**Table 2:** **CSpV1 detection and partial genome sequencing in** ***C. parvum*** **isolates.**

Spl	Year	Dpt	GP60 subtyping	Genbank acc #	RdRpV	VL	Genbank acc #	CPV	VS	Genbank acc #
B36	2020	42	IIaA15G2R1	OQ722152	pos	pos	OQ749437	pos	pos	OQ749467
B38	2021	42	IIaA17G1R1	OQ722153	pos	pos	OQ749438	pos	pos	OQ749468
B40	2021	42	IIaA17G1R1	OQ722172	pos	pos	OQ749439	pos	pos	OQ749469
B41	2021	3	IIaA15G2R1	OQ722154	pos	pos	OQ749440	pos	pos	OQ749470
B42	2021	3	IIaA15G2R1	OQ722155	pos	pos	OQ749441	pos	pos	OQ749471
B43	2021	3	IIaA15G2R1	OQ722156	pos	pos	OQ749442	pos	pos	OQ749472
B44	2021	58	IIaA15G2R1	OQ722157	pos	pos	OQ749443	pos	pos	OQ749473
B45	2021	3	IIaA15G2R1	OQ722158	pos	pos	OQ749444	pos	pos	OQ749474
B46	2021	63	IIaA15G2R1	OQ722159	pos	pos	OQ749445	pos	pos	OQ749475
B47	2021	63	IIaA15G2R1	OQ722160	pos	pos	OQ749446	pos	pos	OQ749476
B48	2021	3	IIaA15G2R1	OQ722161	pos	pos	OQ749447	pos	pos	OQ749477
B49	2021	3	IIaA15G2R1	OQ722162	pos	pos	OQ749448	pos	pos	OQ749478
B50	2021	3	IIaA15G2R1	OQ722163	neg	neg		neg	neg	
B51	2021	63	IIaA15G2R1	OQ722164	pos	pos	OQ749449	pos	pos	OQ749479
B52	2021	63	IIaA15G2R1	OQ722165	pos	pos	OQ749450	pos	pos	OQ749480
B53	2021	3	IIaA15G2R1	OQ722166	pos	pos		neg	pos	OQ749481
B54	2021	57	unknown	-	pos	pos	OQ749451	pos	pos	OQ749482
B55	2021	57	IIaA15G2R1	OQ722167	pos	pos	OQ749452	pos	pos	OQ749483
B65	2022	8	IIaA15G2R1	OQ722168	pos	pos	OQ749434	pos	pos	
B66	2022	8	IIaA15G2R1	OQ722169	pos	pos	OQ749435	pos	pos	
B67	2022	8	IIaA15G2R1	OQ722170	pos	pos	OQ749436	pos	pos	
O1	2018	81	IIaA15G2R1	MN037849	pos	pos	OQ749453	pos	neg	
O10	2019	81	IIaA16G3R1	MN037858	pos	pos		pos	pos	OQ749488
O11	2019	81	IIaA15G2R1	MN037859	pos	pos	OQ749456	pos	pos	OQ749489
O12	2019	81	IIaA15G2R1	MN037860	pos	neg		pos	pos	OQ749490
O13	2019	81	IIaA15G2R1	MN037861	pos	pos		neg	pos	OQ749491
O14	2019	81	IIaA15G2R1	MN037862	pos	pos		neg	pos	OQ749492
O15	2019	81	IIaA15G2R1	MN037863	neg	neg		neg	pos	OQ749493
O16	2019	81	IIaA16G3R1	MN037864	pos	pos	OQ749457	pos	pos	OQ749494
O17	2019	81	IIaA15G2R1	MN037865	neg	neg		neg	pos	
O18	2019	81	IIaA15G2R1	MN037866	pos	pos	OQ749458	pos	pos	OQ749495
O19	2019	81	IIaA15G2R1	MN037867	pos	pos	OQ749459	pos	pos	OQ749496
O2	2018	81	IIaA15G2R1	MN037850	pos	neg		pos	pos	
O20	2019	81	IIaA15G2R1	MN037868	pos	neg		pos	pos	OQ749497
O21	2019	81	IIaA15G2R1	MN037869	pos	pos	OQ749460	pos	pos	OQ749498
O22	2019	81	IIaA15G2R1	MN037870	neg	neg		neg	neg	
O23	2020	45	IIaA17G2R1	MN037871	pos	pos	OQ749461	pos	neg	
O3	2019	87	IIaA15G2R1	MN037851	pos	pos	OQ749454	pos	pos	OQ749484
O4	2019	87	IIaA15G2R1	MN037852	neg	neg		pos	neg	
O5	2019	87	IIaA15G2R1	MN037853	pos	pos	OQ749455	pos	pos	OQ749485
O6	2019	87	IIaA15G2R1	MN037854	pos	neg		pos	pos	OQ749486
O7	2019	87	IIaA15G2R1	MN037855	neg	neg		pos	pos	
O73	2021	64	IIaA15G2R1	OQ722148	pos	pos	OQ749462	pos	pos	OQ749499
O74	2021	46	IIdA18G1R1	OQ722149	pos	pos	OQ749463	pos	pos	OQ749500
O75	2021	46	IIdA18G1R1	OQ722150	pos	pos	OQ749464	pos	pos	OQ749501
O76	2021	46	IIdA18G1R1	OQ722151	pos	pos	OQ749465	pos	pos	OQ749502
O77	2021	46	IIdA18G1R1	OQ747365	pos	pos	OQ749466	pos	pos	
O78	2021	46	IIdA18G1R1	OQ747366	neg	pos		pos	pos	
O79	2021	46	IIaA15G2R1	OQ747367	pos	pos		pos	pos	
O8	2019	87	IIaA15G2R1	MN037856	pos	neg		neg	pos	
O9	2019	87	IIaA15G2R1	MN037857	pos	neg		pos	pos	OQ749487
C1	2019	81	IIaA15G2R1	MN037844	pos	pos		neg	pos	OQ749503
C2	2019	81	IIaA15G2R1	MN037845	pos	neg		neg	pos	
C3	2019	81	IIaA15G2R1	MN037847	pos	neg		neg	pos	OQ749504
C4	2019	81	IIaA15G2R1	MN037848	neg	pos		neg	neg	

^*^Spl: Sample. Letter (B, bovine / cattle; C, Caprine / goats and O, ovine / sheep); Dpt: Department; Cryspovirus PCR was carried out using primers RdRPV and CPV from Jenkins et al. [33] primers VL and VS from Murakoshi et al. [25]. GP60 *C. parvum* subtyping and VL/VS Cryspovirus amplicon were followed with Sanger sequencing. Genbank accession numbers are provided.

### RNA extraction, and Cryspovirus sequencing

Disruption of oocyst walls was achieved using ten freeze–thaw cycles and a proteinase K treatment for 1 h at 55 °C. Total RNA were extracted with RNeasy mini kit (Qiagen) following the manufacturer’s recommendations. Total RNA was used for cDNA synthesis with Maximus H minus reverse transcriptase (Thermofisher). PCR was carried out with primer sets amplifying the RNA-dependent RNA polymerase (RdRP) (dsRNA1: CPVL_ORF_F 5′-AAGTTTGTCAATATCTATGAGATAC-3′, CPVL_ORF_R 5′-TCCATAAATTTTGTGACTCCTG-3′) and capsid (dsRNA2: CPVS_ORF_F 5′-ATTACAAGTTTTGAATCAATAGAG-3′, CPVS_ORF_R 5′-ATGGGAGCGATCTGCGCTACAC-3′) genes as initially described by Murakoshi et al. [25]. The resulting 1468 bp fragments and 867 bp fragments represent 80% of dsRNA1 and 58% of dsRNA2 genome fragments. They were visualized using gel electrophoresis before Sanger sequencing (Genoscreen). Genbank accession numbers of successful sequencing are provided in Table 2. Additional PCR were carried out using a second set of primers amplifying smaller fragments of the RdRP (RdrpV-F: 5′-TGGGCATATGGTGCTCACTA-3’; RdrpV-R: 5′-GCTAAGAGAT CGT AGATGTCCA-3) and capsid (CPV-F: 5′-TGGTTCCGATTTTACCGGAA-3′; CPV-R: 5′-ACGACAATTAGGACTCAAATGACC-3′) as described by Jenkins et al. [33]. CSpV1 was considered detected if at least one of the two PCR approaches returned positive results.

### Full-length genome sequencing

CSpV1 genome was obtained from a pool of samples B44 and B45, using RNA sequencing. cDNA libraries were prepared using an Ion total RNAseq Kit (Life technologies, Carlsbad, CA, USA) according to the supplier’s instructions. The cDNA libraries were sequenced using an Ion Proton Sequencer and an Ion PI Chip v2 (Life technologies).

Sequence reads were cleaned and trimmed for adapter removal using fastP version 0.20.1 [34] and sequence quality was verified using FastQC version 0.11.8 [35]. Reads were assembled with rnaSPADES de novo assembler as implemented in SPAdes assembler version 3.10.0 [36]. Resulting contigs were aligned on local nt database with Megablast version 2.10.1 to identify viral references. Then, both sequence reads and assembled contigs were aligned using Burroughs-Wheeler Aligner (BWA, version 0.7.8) [37] against the CSpV1 strain Iowa genome fragments (NC_038843 dsRNA1 and NC_038844 dsRNA2) and visualized in Integrative Genome Viewer (IGV) [38, 39] to control the quality of the consensus sequence extracted using Samtools pileup version 1.8 [40].

### Phylogenetic analyses

The CSpV1 dsRNA1 and dsRNA2 sequences were aligned against all other CSpV1 sequences known to date. Nucleotide and protein percentage of identity were calculated with Clustal Omega [41]. Phylogenetic analysis were conducted on the full-length open reading frame. Phylogenetic tree were then inferred using the maximum likelihood method implemented in PhyML (version 3.0) [42] using the best-fit model and best of NNI and Subtree Pruning and Regrafting (SPR) branch swapping. Support for nodes on the trees were assessed using an approximate likelihood ratio test (aLRT) with the Shimodaira-Hasegawa-like procedure. Trees generated using the Neighbor–Joining and Maximum Parsimony methods gave identical results.

### Data availability

Sequencing data were submitted to Genbank under Bioproject ID PRJNA947786 using the MIUVIG symbiont-associated package to provide metadata on the host of the virus (*C. parvum*) and on the host of the host (cattle, sheep and goat) [43, 44].

## Results

**Figure 1:**
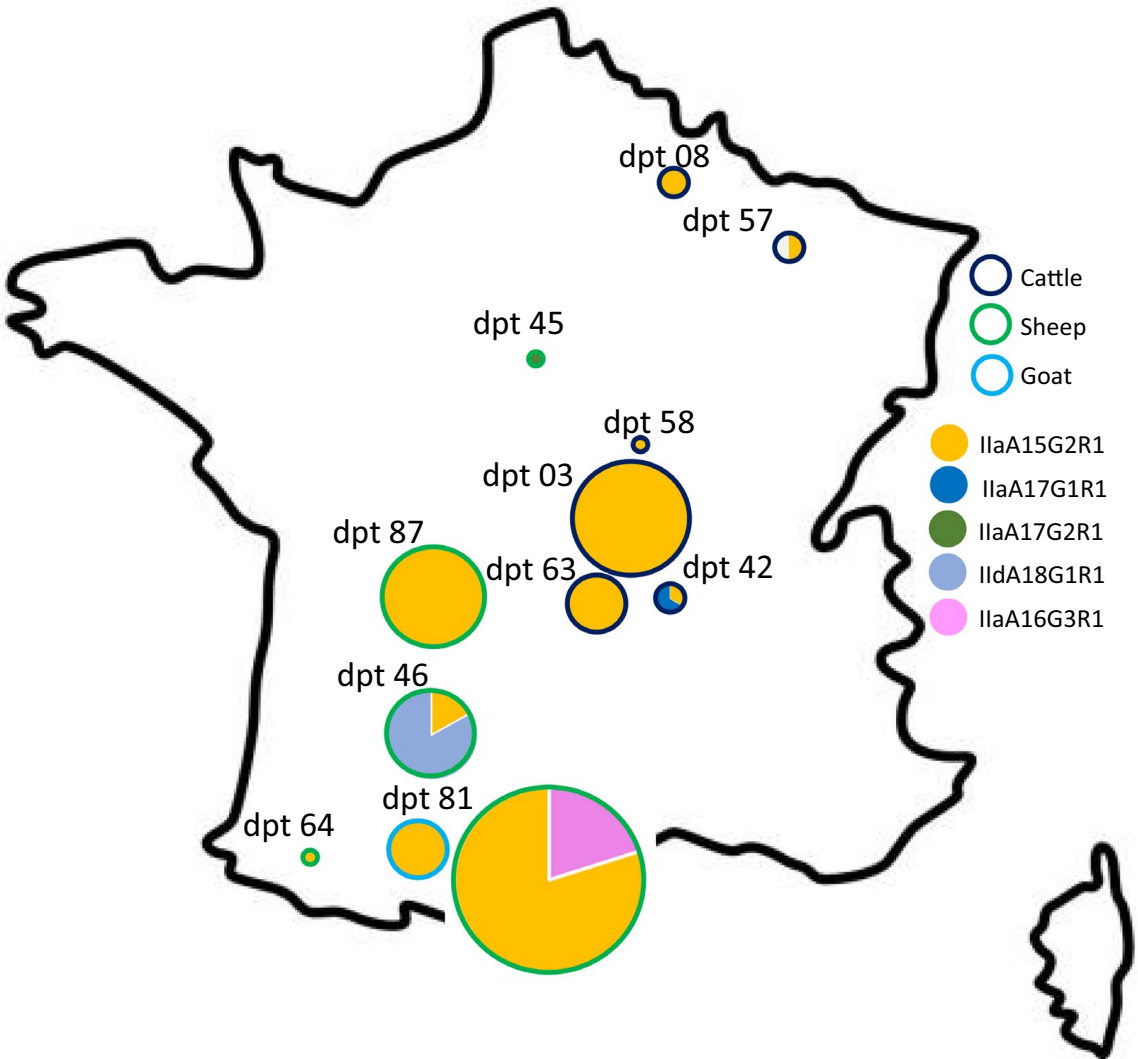
***C. parvum*** **subtyping in France between 2019 and 2021.** *Gp60* based subtyping of *C. parvum* was conducted on isolates from 11 different French departments collected from Cattle, Sheep and Goats. A pie chart is provided for each department (dpt) within which *C. parvum* was detected, and provide the *C. parvum* subtype and the host. The size of the pie charts is proportional to the number of samples collected.

None of the healthy goats, sheep and cattle were infected by *Cryptosporidium*. Among the 57 diseased animals presenting diarrhea, 55 were infected with *Cryptosporidium* and one individual was infected with *Giardia*. 18S PCR and sequencing revealed the presence of *C. parvum* only*.* This was in agreement with previous studies in France showing that *C. parvum* is responsible for most infections in pre-weaned calves, lambs and goats [45]. In France, the two most prevalent *C. parvum* belong to subtype families IIa and IId [14]. For one cattle sample with low parasite load, the 18S gene could not be sequenced. For all other samples, *C. parvum* subtyping revealed that cattle were infected by subtypes IIaA15G2R1 (18 ind; 90%) and IIaA17G1R1 (2 ind, 10%), sheep were infected by subtypes IIaA15G2R1 (22 ind; 73%), IIaA16G3R1 (2 ind; 7%), IIaA17G2R1 (1 ind, 3%) and IIdA18G1R1 (5 ind; 17%), and all 4 goat kids were infected by IIaA15G2R1 (Figure 1, Table 2). Thus, IIaA15G2R1 *C. parvum* subtype was the most prevalent, and was found in 11 departments on all four successive years (2018 to 2022). IIaA17G1R1 and IIaA17G2R1 were found in one department in 2020 and 2021, while IIaA16G3R1 and IIdA18G1R1 were found only once, in one department.

The full-length genome of CSpV1 from France was obtained from a pool of *C. parvum* of subtype IIaA15G2R1 collected from cattle in 2021 (Samples B44 and B45; Genbank acc numbers OQ686777 and OQ686778 for dsRNA1 and dsRNA2 respectively). The representative dsRNA1 and dsRNA2 FR strain genome was obtained with a coverage depth of 50 and 70 reads per position and with 0.6 and 0.8% iupac codes for dsRNA1 and dsRNA2, respectively. The genome sequence was compared to the three available full-length Cryspovirus genomes from Iowa (Iowa), Kansas (KSU) and China. The FR strain dsRNA1 shows a maximum of 97.3% nucleotide (nc) identity to the Iowa strain and a minimum of 96.4% nc identity to the KSU strain from North America. The RdRP protein shows 99% and 97% amino-acid (aa) identity to the Iowa and KSU strains, respectively. The FR strain dsRNA2 shows a maximum of 98.4% nc identity to the Iowa strain and a minimum of 98% nc identity to KSU strain and was more distantly related to the related viruses discovered in *Cryptosporidium meleagridis* (85.6% nc identity), *Cryptosporidium felis* (86.7% nc identity), and *Cryptosporidium hominis* (90.8% nc identity). The capsid protein shows 98.6% and 99.1% aa identity to the Iowa and KSU strains, respectively.

**Figure 2:**
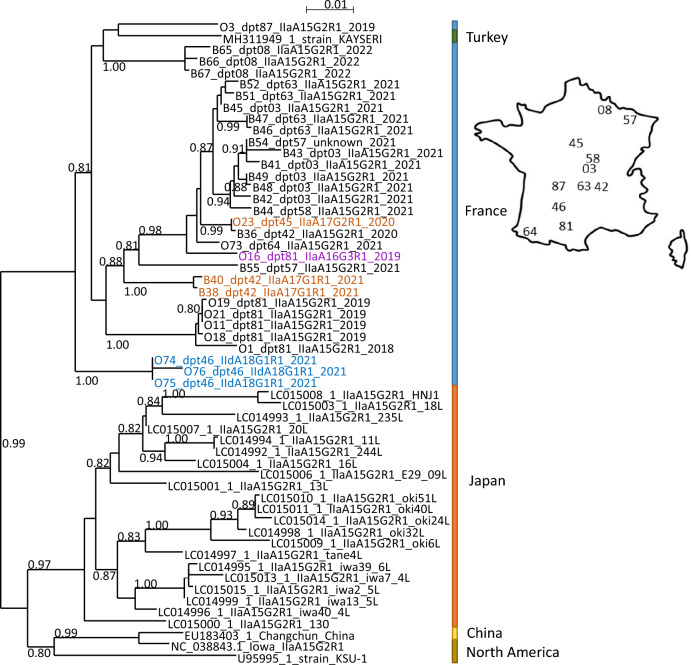
**Tracking CSpV1 movements and evolution through phylogenetic analysis of dsRNA1.** Phylogenetic tree of a 1468 nc long fragment of dsRNA1 of CSpV1 using all available CSpV1 sequences. The tree was inferred in PhyML using the LG substitution model. Branch points indicate that the results of Shimodaira-Hasgawa branch test > 0.8. Scale bar shows the number of nucleotide changes. Genbank virus isolate names are given as follows: accession number_*C. parvum* subtype_strain name. French virus isolate names are provided as follows: Letter (B: bovine/cattle and O; ovine / sheep) followed with individual number, department number, *C. parvum* subtype, and sampling year. A map of France providing the localisation of departments (dpt) is provided next to the tree.

**Figure 3:**
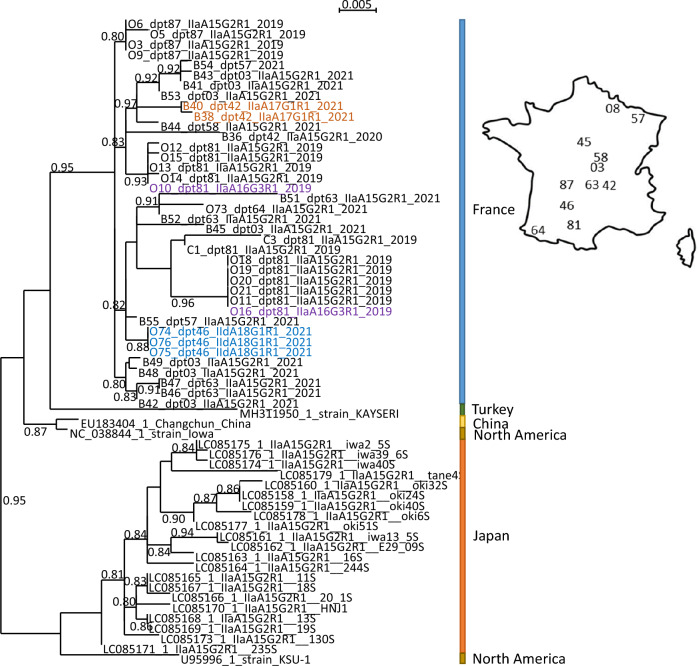
**Tracking CSpV1 movements and evolution through phylogenetic analysis of dsRNA2.** Phylogenetic tree of a 867 nc long fragment of dsRNA2 of CSpV1 using all available CSpV1 sequences. The tree was inferred in PhyML using the LG substitution model. Branch points indicate that the results of Shimodaira-Hasgawa branch test > 0.8. Scale bar shows the number of nucleotide changes. Genbank virus isolate names are given as follows: accession number_*C. parvum* subtype_strain name. French virus isolates names are provided as follows: Letter (B: bovine / cattle, C, Caprine / goats and O; ovine / sheep) followed with individual number, department number, *C. parvum* subtype, and sampling year. A map of France providing the localisation of departments (dpt) is provided next to the tree.

Using two different sets of primers per virus genome fragment, we confirmed the presence of CSpV1 within all but two isolates of *C. parvum* (Table 2), including in the sample for which the *C. parvum* subtype could not be determined. This high prevalence was in accordance with previous reports [21, 25]. Good quality partial sequences of CSpV1 virus dsRNA1 encoding the RdRP, and dsRNA2 encoding the Capsid proteins were obtained for 30 and 42 samples, respectively (Genbank accession numbers are provided in Table 2). CSpV1 partial genome sequencing provided evidence of co-infection by two different genotypes of CSpV1 in *C. parvum* isolate O77, depicted by the presence of two overlapping peaks on chromatograms. Interestingly, point variations observed co-localize with nucleotide positions that show variations when comparing all sequences with each other, further supporting the co-infection hypothesis (Additional file 1). A greater genetic diversity was observed within dsRNA1 that shows an average of 95.6% nc identity to CSpV1 strain Iowa whereas dsRNA2 presents an average of 98% nc identity to CSpV1 strain Iowa. We conducted phylogenetic analyses on the nucleotide sequences of dsRNA1 and dsRNA2 and yielded similar results. As previously described, all sequences from Japan clustered closely together on a single branch. Similarly, sequences from France clustered closely together and are most closely related to the only other CSpV1 partial virus sequence from the European continent (origin: Turkey) (Figures 2 and 3).

## Discussion

The parasite population structure and rate of CSpV1 vertical and horizontal transmission is expected to influence virus evolution. Here, phylogenetic analyses conducted on both genome fragments (dsRNA1 and dsRNA2) show that CSpV1 from France clustered together with the sequence of CSpV1 from Turkey, and on a branch distinct from CSpV1 collected from North America or Asia (Figures 2 and 3). This result indicates that the CSpV1 dsRNA1 and dsRNA2 can distinguish sampling area at the continental level. Furthermore, we provide herein the first characterization of CSpV1 from different subtypes of *C. parvum*. Indeed, studies conducted on CSpV1 from North America and Japan focused on *C. parvum* subtype IIaA15G2R1 [25, 46], while the subtype of *C. parvum* from which the CSpV1 from Turkey and China were sequenced had not been characterized [47, 48]. The fact that regardless of the subtype of *C. parvum* (IIaA15G2R1, IIaA17G2R1 and IIdA18G1R1), all European sequences cluster together indicates that CSpV1 host shift between different parasite subtypes does occur. The rate of such host shift might be high considering that, focusing on sequencing from France only, we did not observe any clustering of CSpV1 depending on parasite subtypes. Experimental studies would be needed to estimate the rate of virus host shift and characterize the underlying mechanism. Host shift can only occur within a host co-infected by two subtypes of *C. parvum*. Coinfections at the species level have frequently been reported and recent studies have demonstrated that different *C. parvum* subtypes recombine and can give rise to more highly virulent progeny [49, 50]. Indeed, recent reports indicate that classical methods based on Sanger sequencing of PCR amplified *gp60* alleles strongly underestimate the intra-host genotype diversity [51–53]. Moreover, single oocysts can harbor a mixed population of sporozoites, which provide opportunities for CSpV1 host shift between *gp60* subtypes of *C. parvum* in the same animal. Host co-infection by different strains of *C. parvum* could also explain why we found some evidence of co-infection by different CSpV1 in isolate O77. We did not conduct clonal isolation of *C. parvum*, which means that if the host was co-infected by different *C. parvum* strains, they were present in the mixture that was analyzed. Therefore, the working hypothesis that each *C. parvum* strain carries only a single CSpV1 remains to be tested through more advanced analyses of CSpV1 genotype and *C. parvum* genotyping.

It has been suggested that CSpV1 evolution can be used to track movement of *C. parvum* [25]. Thus, we compared the phylogenetic position of CSpV1 dsRNA1 and dsRNA2 with the ruminant host, sampling year, and geographic locations. Our results indicate that samples collected on a given year and given location are more likely to host the same subtype of *C. parvum* and the same CSpV1 strain (Figures 1 and 2). In addition, when sampling occurred on successive years in the same location, we found the same subtype of *C. parvum* and the same CSpV1 strain (Figure 2). Yet, there is no distinct clustering of viruses per department or ruminant host: closely related CSpV1 were found in distant departments (i.e. viruses of IIaA15G2R1 from cattle of departments 63, 03, 57 and 58, Figures 1 and 2); also, closely related CSpV1 are often found in different host species (Figures 2 and 3). Our results point towards (i) a close association between CSpV1movement and *C. parvum* movement, (ii) recent migrations of *C. parvum* among distantly located departments and (iii) incidental transmission of *C. parvum* between ruminants. Inter-species local transmission of *Cryptosporidium* spp*.* is characteristic of the parasite epidemiology [54–56]. Thus, the presence of *C. parvum* in many animal species, inter-species transmission, movements of animals due to trade among different regions of the country, and the potential role of wild animals in parasite dispersion [56], implies that the sources of *C. parvum* and CSpV1 movements are multiple.

In conclusion, we provide the first genomic data of CSpV1 in France. We also provide the first comparative analysis of the genome of CSpV1 from different *C. parvum* subtypes, from different ruminant hosts and over successive years. This analysis provides insightful information regarding both *C. parvum* and CSpV1 transmission and evolution. In agreement with Murakoshi et al. [25], our results suggest that CSpV1 is vertically transmitted in *C. parvum*, and that transmission between subtypes likely occurs as a result of recombination during the parasite’s sexual life-cycle. While gp60 gene coding is the most common marker used for epidemiological surveillance of *C. parvum*, the limitations associated with this approach are recognized and novel techniques such as whole genome sequencing and multilocus genotyping are being investigated as alternative approaches [49, 57]. Herein, we identified CSpV1 in all but two *C. parvum* isolates, which can either suggest the absence of the virus within these two *C. parvum*-positive samples, or illustrate that our sensitivity was suboptimal. Given that CSpV1 was often identified using a set of primers and not others suggest that neither of our primers provided sufficient sensitivity. Moreover, we failed at obtaining good quality CSpV1 sequences from many samples, which limited the scope of our analyses. In the future, amplicon-based sequencing approaches are likely to provide better sensitivity, while allowing for the sequencing of multiple variants of CSpV1 within the same sample. CSpV1 genome sequencing may represent a low-cost alternative approach to track *C. parvum* epidemiology and will help identify co-infections, and characterize the rates of outcrossing and inter-species transmission. Coupling amplicon-based sequencing of CSpV1 with multilocus genotyping of *C. parvum* would most likely provide a more accurate description of the parasite and virus co-diversification. Whether the virus’ ability to jump host between *C. parvum* belonging to different subclades can modulate the protozoan parasite’s ability to infect different ruminants, its pathogenicity, and zoonotic potential remains to be investigated through experimental approaches.

### Supplementary Information

**Additional file 1. Coinfection of O77 by different variants of CSpV1.** **A** Example of point mutation in capillary sequencing chromatogram confirming the presence of different variants of CSpV1 in sample O77. **B** Alignment of all sequences to the same region showing that the point mutation co-localize with a region where variation occur often between different CSpV1.
