# Supplementary figures and images for: First identification of Cryptosporidium parvum virus 1 (CSpV1) in various subtypes of Cryptosporidium parvum from diarrheic calves, lambs and goat kids from France
Source: Vet Res. 2023 Aug 22;54:66. doi: 10.1186/s13567-023-01196-4 (PMC10464362; doi:10.1186/s13567-023-01196-4)

## Slide 1
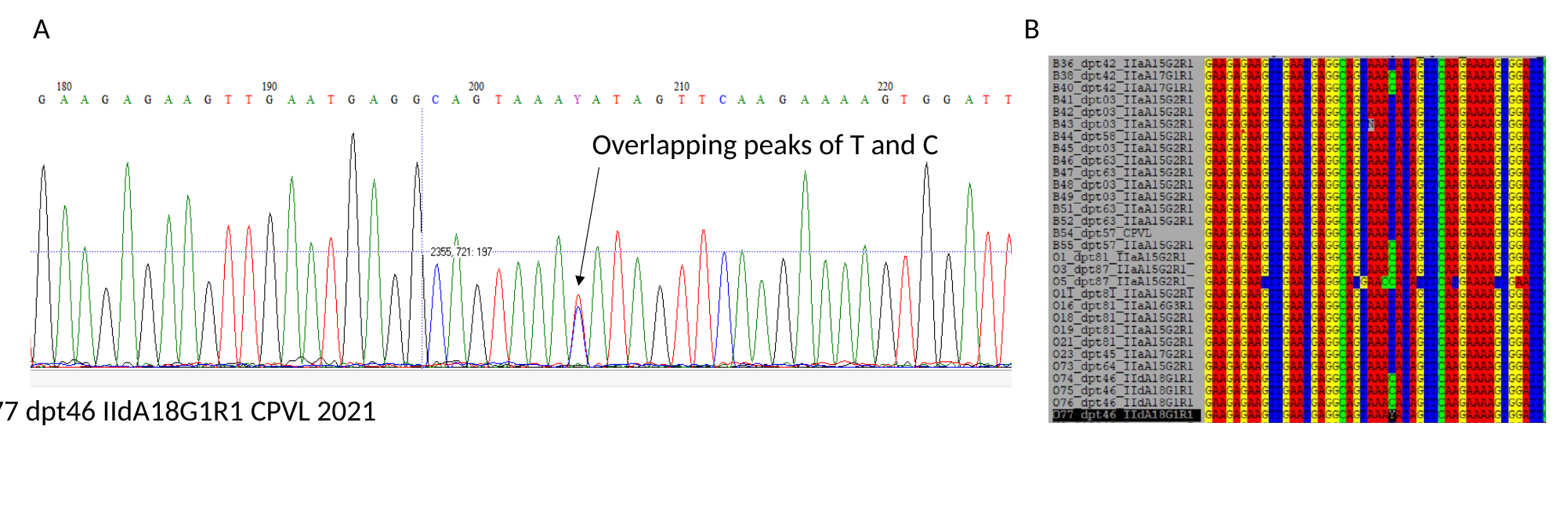

A
B
Overlapping peaks of T and C
O77 dpt46 IIdA18G1R1 CPVL 2021

Supplement: Supplementary file 1 — Additional file 1. Coinfection of O77 by different variants of CSpV1. A Example of point mutation in capillary sequencing chromatogram confirming the presence of different variants of CSpV1 in sample O77. B Alignment of all sequences to the same region showing that the point mutation co-localize with a region where variation occur often between different CSpV1. [file 13567_2023_1196_MOESM1_ESM.pptx]
